# Bufalin Induces Apoptosis of Human Osteosarcoma U-2 OS Cells through Endoplasmic Reticulum Stress, Caspase- and Mitochondria-Dependent Signaling Pathways

**DOI:** 10.3390/molecules22030437

**Published:** 2017-03-10

**Authors:** Ching-Hsiao Lee, Yung-Luen Shih, Mei-Hui Lee, Man-Kuan Au, Yung-Liang Chen, Hsu-Feng Lu, Jing-Gung Chung

**Affiliations:** 1 Department of Medical Technology, Jen-Teh Junior College of Medicine, Nursing and Management, Miaoli Country 356, Taiwan; heecs@ms38.hinet.net; 2 Department of Pathology and Laboratory Medicine, Shin Kong Wu Ho-Su Memorial Hospital, Taipei 111, Taiwan; t005524@ms.skh.org.tw; 3 School of Medical Laboratory Science and Biotechnology, Taipei Medical University, Taipei 110, Taiwan; 4 School of Medicine, College of Medicine, Fu-Jen Catholic University, New Taipei City 242, Taiwan; 5 Department of Genetic Counseling Center, Changhua Christian Hospital, Changhua 500, Taiwan; 29561@cch.org.tw; 6 Department of Orthopedics, Cheng Hsin General Hospital, Taipei 112, Taiwan; ch6215@chgh.org.tw; 7 Department of Medical Laboratory Science and Biotechnology, Yuanpei University, Hsinchu 300, Taiwan; yunliang@mail2000.com.tw; 8 Restaurant, Hotel and Institutional Management, Fu-Jen Catholic University, New Taipei City 242, Taiwan; 9 Department of Clinical Pathology, Cheng Hsin General Hospital, Taipei 112, Taiwan; 10 Department of Biological Science and Technology, China Medical University, Taichung 404, Taiwan; 11 Department of Biotechnology, Asia University, Wufeng, Taichung 413, Taiwan

**Keywords:** bufalin, human osteosarcoma U-2 OS cells, endoplasmic reticulum stress-dependent signaling pathways, caspase-dependent signaling pathways, mitochondria-dependent signaling pathways

## Abstract

Bone cancer is one of the cancer-related diseases, and there are increased numbers of patients with bone cancer worldwide. Therefore the efficacy of treatment of bone cancer is considered extremely vital. Bufalin has been showed to have biological activities including anticancer activities in vitro and in vivo. However, the exact associated mechanisms for bufalin induced apoptosis in human bone cancer cells are still unclear. In the present study, we investigated the effect of bufalin on the cytotoxic effects in U-2 OS human osteosarcoma cells. For examining apoptotic cell deaths, we used flow cytometry assay, Annexin V/PI double staining, and TUNNEL assay. Reactive oxygen species (ROS), Ca^2+^, mitochondrial membrane potential (ΔΨ_m_), and caspase-8, -9 and -3 activities were measured by flow cytometry assay. Furthermore, western blotting and a confocal laser microscopy examination were used for measuring the alterations of apoptotic associated protein expression and translocation, respectively. The results indicated that bufalin induced cell morphological changes, decreased the viable cell number, induced apoptotic cell death, and increased the apoptotic cell number, and affected apoptotic associated protein expression in U-2 OS cells. Bufalin increased apoptotic proteins such as Bak, and decreased anti-apoptotic proteins such as Bcl-2 and Bcl-x in U-2 OS cells. Furthermore, bufalin increased the protein levels of cytochrome c (Cyto c), AIF (Apoptosis inducing factor) and Endo G (Endonuclease G) in cytoplasm that were also confirmed by confocal microscopy examination. Based on those findings, bufalin induced apoptotic cell death in U-2 OS cells may be via endoplasmic reticulum (ER) stress, caspase-, and mitochondria-dependent pathways; thus, we may suggest that bufalin could be used as an anti-cancer agent for the treatment of osteosarcoma in the future, and further in vivo studies are needed.

## 1. Introduction

The primary bone tumors account in all cancers less than 0.2%. However, bone is a common site for the development of tumor metastases [1], and the main type of primary bone tumors are osteosarcomas [2] which arise from osteoid tissue and are classified as a malignant mesenchymal neoplasm [1]. The prevalence rate is 0.9 per 100,000 persons per year for all bone and joint cancers [2,3,4] based on 2016 reports from The American Cancer Society’s estimates, according to which there are 3300 new cases and 1490 deaths from these cancers [3,4]. In Taiwan, bone cancer is the 27th most common cancer based on a report in 2016 from the Department of Health (Taiwan), which indicated that the bone cancer mortality rate was 0.4 per 100,000 people [5]. Currently, the treatments for bone cancer are surgery, radiotherapy, and chemotherapy or a combination of those treatments. The therapies have been shown to affect the quality of life of survivors with osteosarcoma [6,7]. Therefore, the development of oral chemotherapeutics has gained attention for improving the survival and quality of life of a large number of patients with osteosarcoma [8]. Studies have focused on the compounds from natural products for osteosarcoma cancer patients [9].

The best strategy for anticancer drugs is to induce cancer cell apoptosis; thus, the induction of apoptosis also could be a promising strategy to improve radiation-induced tumor cell death [10]. Apoptosis (programmed cell death) is one of the important results from anticancer drug induced cytotoxicity. It is well documented that the characteristics of cell apoptosis that are facilitated by a series of gene regulatory cell-signaling pathways, such as the perturbation of mitochondrial function, have been observed in the apoptotic cascade [11,12]. Numerous studies have shown that anticancer drugs may damage the mitochondria by increasing the permeability of the outer mitochondrial membrane and then affect the mitochondrial membrane potential (ΔΨ_m_) [13,14,15]. After the occurrence of dysfunction of mitochondria that leads to the cytochrome c release and to the activation of caspases for causing apoptosis, AIF (Apoptosis inducing factor) and Endo G (Endonuclease G) are released from themitochondria to the activation of nucleases, causing apoptosis through the caspases-apoptotic pathway, which can thus be divided into caspase- and mitochondria-dependent signaling pathways [16,17]. 

Bufalin, the major digoxin-like immunoreactivity component, is one of the components of bufadienolides found in Chan Su [18] and has been shown to suppress cell proliferation and induce apoptosis in lung cancer [19], melanoma [20], breast cancer [21], hepatocellular carcinoma [22], gastric cancer [23], and glioma cells [24] and in rheumatoid arthritis fibroblast-like synoviocytes [25]. Recently, in our laboratory, we found that bufalin induced DNA damage in NCI-H460 cells and also inhibited its DNA repair and checkpoint function [26]. Numerous studies have shown that bufalin induced cytotoxic effects through the induction of apoptosis in many human cancer cells including human osteosarcoma U-2 OS cells [27,28,29], human osteosarcoma MG-63 cells [30], and both MTX-sensitive and MTX-resistant human osteosarcoma U-2 OS cells [31]; however, the possible molecular mechanisms of bufalin induced cytotoxic cell death involving endoplasmic reticulum (ER) stress in human osteosarcoma is still unclear. The possible molecular mechanisms of bufalin induced cytotoxic cell death involving endoplasmic reticulum (ER) stress in human osteosarcoma is still unclear; thus, we investigate the anticancer potential of bufalin against human osteosarcoma U-2 OS cells in vitro. Herein, we demonstrate bufalin induced cell death via cell cycle arrest and the induction of apoptosis in U-2 OS cells via endoplasmic reticulum (ER) stress, caspase-, and mitochondria-dependent pathways.

## 2. Results

### 2.1. Bufalin Induced Cell Morphological Changes and Decreased Total Viability of U-2 OS Cells

**Figure 1:**
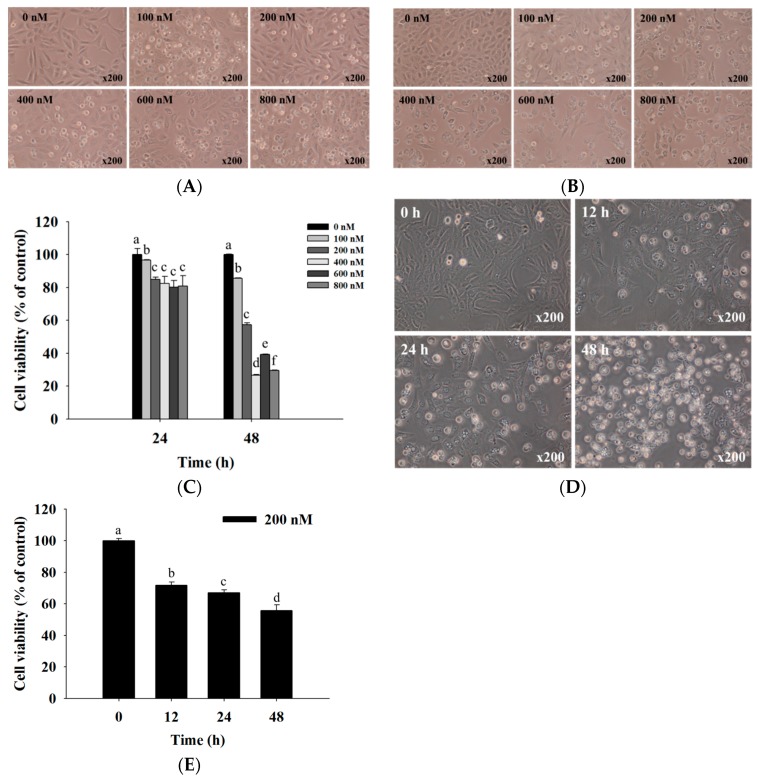
Bufalin induced cell morphological changes and decreased the viability of U-2 OS cells. Cells were treated with 0, 100, 200, 400, 600, and 800 nM of bufalin for (**A**) 24 h and (**B**) 48 h before the cells were examined and photographed for cell morphological changes by contrast-phase microscopy at ×200 and (**C**) were harvested for total percentage of viable cells measurements; or (**D**) were treated with 200 nM bufalin for various time periods before the cells were examined and photographed for cell morphological changes by contrast-phase microscopy at ×200; and (**E**) were harvested for total percentage of viable cells measurements. Morphological changes and total viable cells were observed by flow cytometry, as described in Materials and Methods. Different letters mean significant difference between bufalin-treated groups and the control as analyzed by one-way analysis of variance (ANOVA), followed by Duncan’s multiple range test (DMRT).

U-2 OS cells were exposed to various concentrations of bufalin for various time periods; then we examined the cytotoxic effects, and the results are shown in Figure 1. The results showed that bufalin induced cell morphological changes over 24 (Figure 1A) and 48 h (Figure 1B) and reduced U-2 OS total cell viability dose- and time-dependently (Figure 1C), with an IC_50_ of 200 nM at 48 h. Figure 1D,E indicated that 200 nM bufalin induced cell morphological changes and reduced the total cell viability time-dependently. Therefore, we selected the measure of 200 nM for further experiments throughout whole text. Based on these results, it was indicated that bufalin induced cell morphological changes and decreased the number of viable U-2 OS cells in vitro.

### 2.2. Bufalin Induced Cell Cycle Arrest in U-2 OS Cells

**Figure 2:**
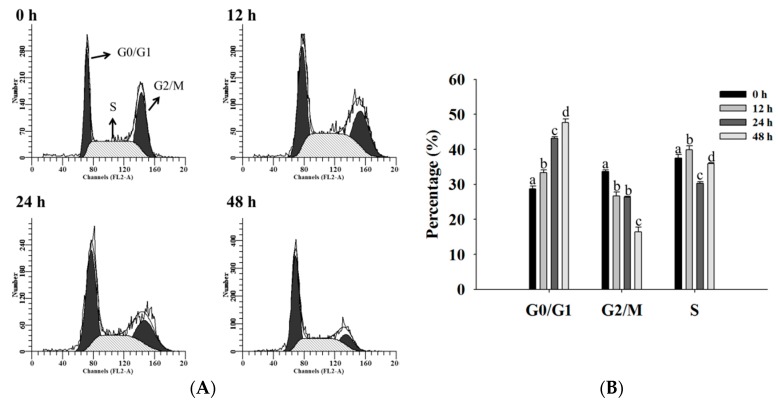
Bufalin induced cell cycle arrest in U-2 OS cells. Cells were treated with 200 nM of bufalin for 0, 12, 24, and 48 h and then were stained with PI (Propidium iodide) for cycle distribution by flow cytometric assay, as described in Materials and Methods. (**A**) Representative profiles and (**B**) percentage of cell cycle distribution. Different letters mean significant difference between bufalin-treated groups and the control as analyzed by one-way analysis of variance (ANOVA), followed by Duncan’s multiple range test (DMRT).

After U-2 OS cells were exposed to bufalin (200 nM) for 0, 12, 24, and 48 h, the cells were collected for cell distribution assay by flow cytometer, and the results are shown in Figure 2. The results indicated that bufalin induced G0/G1 phase arrest and decreased the G2M phase in U-2 OS cells, and these effects are time-dependent.

### 2.3. Bufalin Induced Apoptosis in U-2 OS Cells

**Figure 3:**
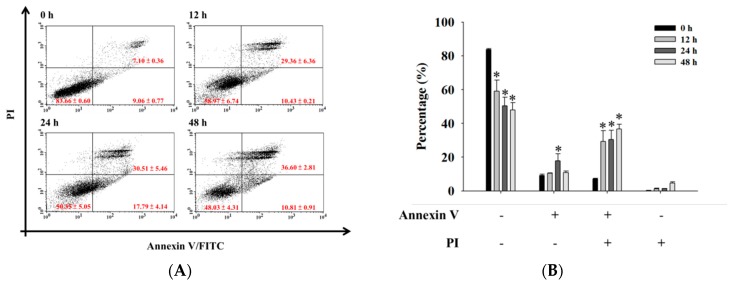

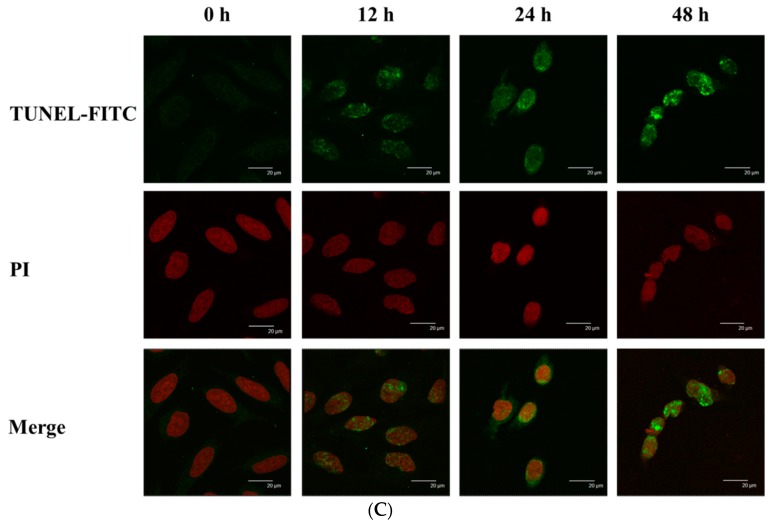
Bufalin induced apoptosis of U-2 OS cells. Cells were treated with 200 nM of bufalin for 0, 12, 24, and 48 h before the cells were (**A**,**B**) double stained using Annexin V/PI and were analyzed by flow cytometry or (**C**) were assayed for TUNNEL assay, as described in Materials and Methods. * *p* < 0.05, significant difference between bufalin-treated groups and the control as analyzed by Student’s *t* test.

For further confirmation that bufalin decreased the cell number of viable U-2 OS cells via the induction of apoptosis, cells were exposed to bufalin (200 nM) for 0, 12, 24, and 48 h, were double stained by Annexin V/PI, and were analyzed by flow cytometry. All results are shown in Figure 3A,B. Alternately, cells were harvested and examined by TUNNEL assay, and the results are shown in Figure 3C. The results indicated that bufalin induced earlier and late apoptotic cell death in U-2 OS cells. These effects are significant (* *p* < 0.05).

### 2.4. Bufalin Induced Reactive Oxygen Species and Ca^2+^ Productions and Decreased the Levels of Mitochondrial Membrane Potential (*ΔΨ_m_*) in U-2 OS Cells

**Figure 4:**
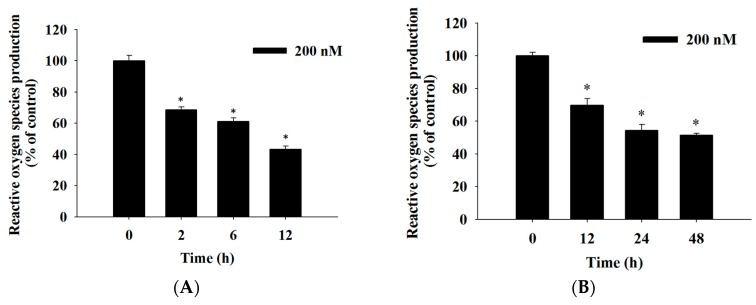

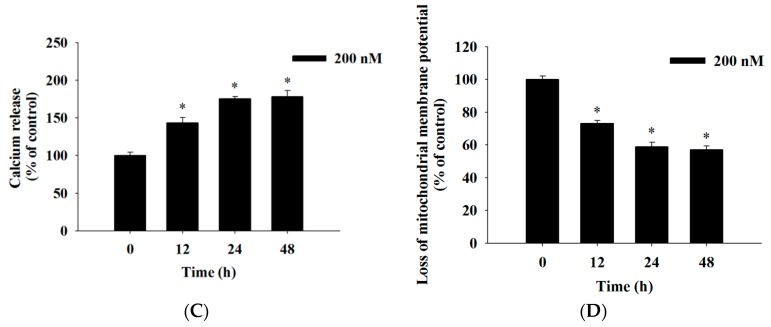
Bufalin induced reactive oxygen species and Ca^2+^ production and decreased the levels of mitochondrial membrane potential (ΔΨ_m_) in U-2 OS cells. Cells (1 × 10^5^ cells/well) were treated with bufalin (200 nM) for various time periods. Cells were isolated and were re-suspended in 500 μL of DCFH-DA (2′,7′-Dichlorofluorescin diacetate) (10 μM) for further ROS (Reactive oxygen species) (H_2_O_2_) (**A**: short time, **B**: long time), (**C**), re-suspended in 500 μL of Fluo-3/AM (2.5 μg/mL) for further intracellular Ca^2+^ concentrations, and (**D**) re-suspended in 500 μL of DiOC_6_ (4 μmol/L) for 30 min for further levels of ΔΨ_m_ measurement, as described in Materials and Methods. The results are shown as a mean ± SD (*n* = 3); * *p* < 0.05, significant difference between bufalin-treated groups and the control as analyzed by Student’s *t* test.

For further understanding of bufalin induced cell apoptosis in U-2 OS cells and whether it involved the production of ROS (Reactive oxygen species) and Ca^2+^ or a dysfunction of mitochondria, the cells were incubated with 200 nM bufalin for 0, 2, 4, 6, 12, 24, and 48 h and were analyzed by flow cytometric assay. The results are shown in Figure 4. The results showed that bufalin decreased ROS production with treatment from 2–48 h (Figure 4A,B) and increased Ca^2+^ production from 12–48 h (Figure 4C). However, this decreased levels of ΔΨ_m_ with 12–48 h treatment (Figure 4D). Based on these findings, it showed that ROS, Ca^2+^ and ΔΨ_m_ are involved in bufalin induced apoptotic cell death in U-2 OS cells in vitro.

### 2.5. Bufalin Increased the Activities of Caspase-3, -9 and -8 in U-2 OS Cells

**Figure 5:**
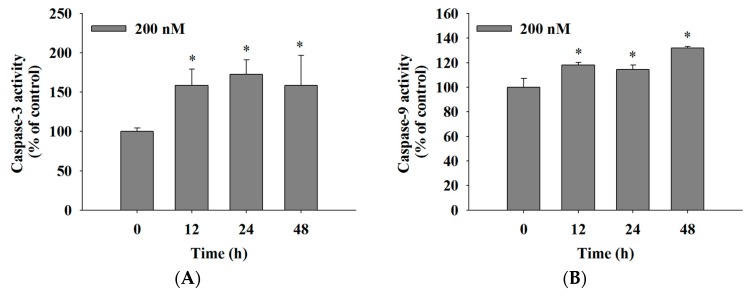

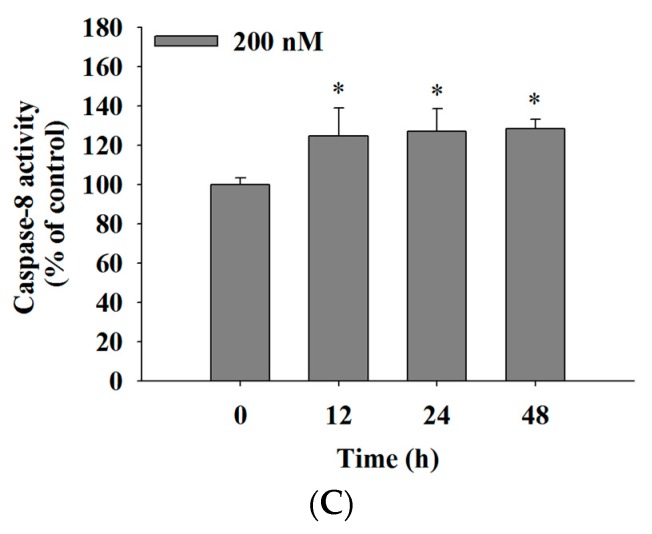
Bufalin affects the activities of caspase-3, -9, and -8 in U-2 OS cells. U-2 OS cells (1 × 10^5^ cells/well) were incubated with 200 nM of bufalin for 0, 12, 24, and 48 h; then the cells were harvested and re-suspended in 25 μL of 10 μM substrate solution containing (**A**) PhiPhiLux-G1D1 for caspase-3, (**B**) CaspaLux9-M1D2 for caspase-9, and (**C**) CaspaLux8-L_1_D_2_ for caspase-8 activity measurements, as described in Materials and Methods. The results are shown as a mean ± SD (*n* = 3); * *p* < 0.05, significant difference between bufalin-treated groups and the control as analyzed by Student’s *t* test.

For further understanding of whether bufalin induces cell apoptosis in U-2 OS cells and whether this involved the activation of caspase-3, -9, and -8, cells were incubated with 200 nM bufalin for 0, 12, 24, and 48 h, and the cells were analyzed by flow cytometric assay. The results are shown in Figure 5. The results indicated that bufalin increased caspase-3 (Figure 5A), -9 (Figure 5B), and -8 (Figure 5C) activities from 12–48 h treatment. These results indicated that bufalin induced cell apoptosis through the activation of caspase-3, -9, and -8 in U-2 OS cells in vitro.

### 2.6. Bufalin Altered Apoptosis Associated Protein Expression in U-2 OS Cells

**Figure 6:**
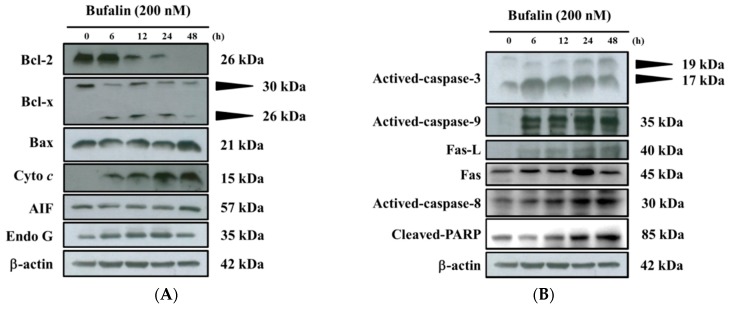

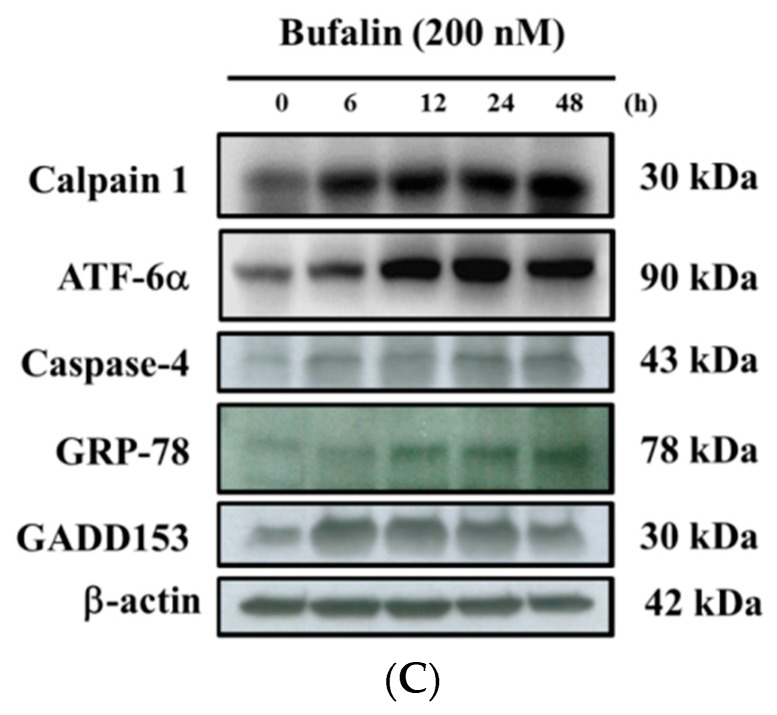
Bufalin affects apoptosis associated protein expression in U-2 OS cells. Cells were treated with 200 nM of bufalin for 0, 6, 12, 24, and 48 h, and then the total proteins were quantitated and the apoptosis associated proteins were measured by Western blotting, as described in Materials and Methods. (**A**) Bcl-2, Bcl-xl, Bax, cytochrome c, AIF and Endo G; (**B**): caspase-8, -9 and -3, Fas, Fas-L, and cleaved PARP (poly (ADP-ribose) polymerase); (**C**): Calpain 1, ATF-6α, Caspase-4, GRP-78 and GADD153.

In order to check whether bufalin induced cell apoptosis of U-2 OS cells is involved in the altered apoptosis associated protein, the cells were incubated with bufalin (200 nM) for 6, 12, 24, and 48 h, and then apoptosis associated proteins were examined and quantitated with Western blotting; the results are shown in Figure 6. The results demonstrated that bufalin significantly increased the expression of cytochrome c, Bax, Endo G, and AIF (Figure 6A); activated-caspase-3, -8, and -9, Fas-L, Fas; and cleaved-PARP (poly (ADP-ribose) polymerase) (Figure 6B); Calpain 1, ATF-6α, caspase-4, GRP-78, and GADD153 (Figure 6C). However, bufalin significantly reduced the expression of anti-apoptotic proteins such as Bcl-x (30 kDa) and Bcl-2 (Figure 6A). Those results indicated that bufalin induced the apoptosis of U-2 OS cells through mitochondria-, ER-, and caspase-dependent pathways.

### 2.7. Bufalin Altered the Translocation of Apoptotic Associated Proteins in U-2 OS Cells

**Figure 7:**
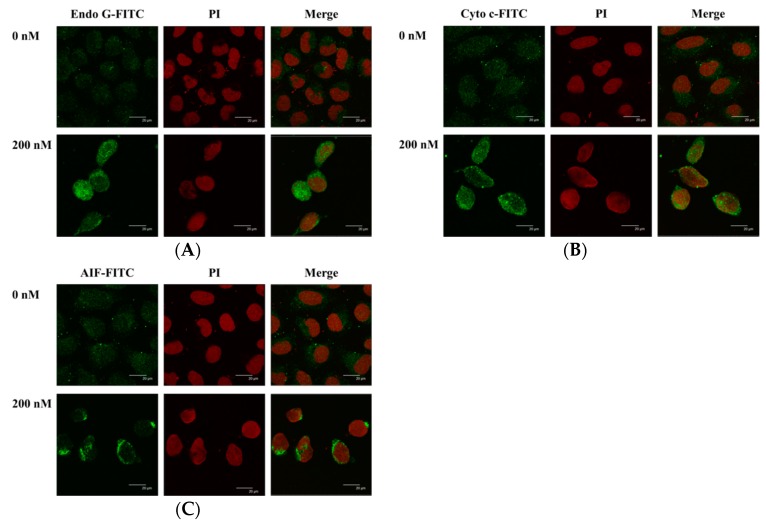
Bufalin affects the translocation of apoptotic-associated proteins in U-2 OS cells. Cells were treated with 200 nM of bufalin for 48 h, and cells were stained by (**A**) anti-Endo G, (**B**) -cytochrome c, and (**C**) -AIF and then were stained with a secondary antibody and were examined and photographed by a Leica TCS SP2 confocal laser microscopic system, as described in Materials and Methods.

In order to further confirm that bufalin affects the translocation of Endo G and that cytochrome c and AIF are involved in apoptosis of U-2 OS cells, cells were incubated with or without 200 nM of bufalin for 48 h and were stained by anti-Endo G, -Cyto c (cytochrome c), and -AIF and then examined and photographed by confocal laser microscopic systems. The results are shown in Figure 7. The results showed that bufalin increased Endo G (Figure 7A), Cyto c (Figure 7B), and AIF (Figure 7C) releases from mitochondria in cytoplasm when compared to the control group.

## 3. Discussion

**Figure 8:**
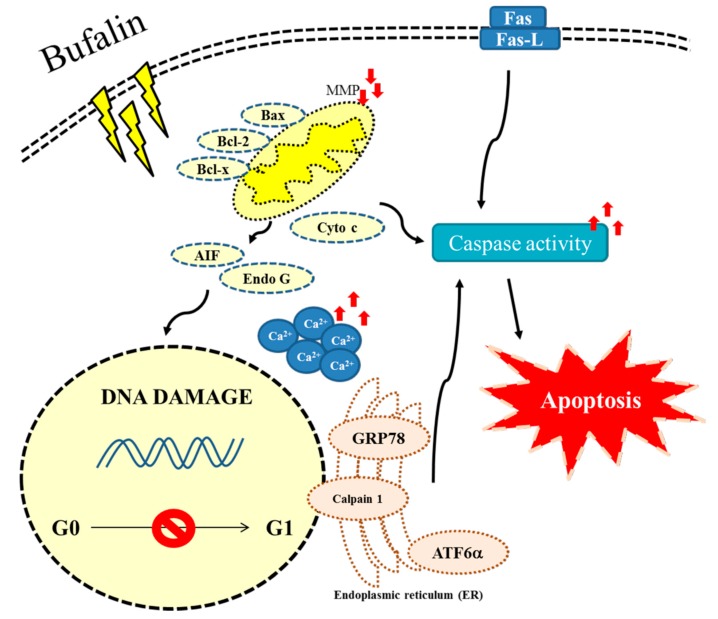
The possible extrinsic and intrinsic signaling pathways for bufalin induced apoptosis in U-2 OS human osteosarcoma cancer cells.

Numerous studies have shown that bufalin has biological activities, including an anticancer function in vitro on many human cancer cell lines through induced cell cycle arrest and cell apoptosis [18,19,20,21,22,23,24,25]. Bufalin has been shown to inhibit the growth and induced apoptosis in both methotrexate (MTX)-sensitive and (MTX)-resistant human osteosarcoma U-2 OS cells [27] and to down-regulate Hsp27 in bufalin-induced apoptosis in osteosarcoma cells [32]. Bufalin inhibited cell growth via the down-regulation of Bcl-2/Bax and the triggering of the mitochondrial pathway in human osteosarcoma MG-63 cells [31]. Recently, it was reported that bufalin induced apoptosis in the U-2 OS human osteosarcoma cell line via triggering of the mitochondrial pathway [28]. However, the molecular mechanisms for the effects of bufalin on human osteosarcoma U-2 OS cells OS remain poorly understood. Therefore, in the present study, we investigate the cytotoxic effects of bufalin on human osteosarcoma U-2 OS cells in vitro. We found that (1) bufalin induced cell death accompanies cell morphological changes and decreases the percentage of the viable cell number (Figure 1); (2) bufalin induced G0/G1 phase arrest and decreased the G2/M phase in U-2 OS cells (Figure 2); (3) bufalin induced cell apoptosis, which was examined by Annexin V/PI double staining and TUNNEL assay (Figure 3A–C); (4) bufalin decreased the production of ROS and the levels of ΔΨ_m_ but increased Ca^2+^ levels (Figure 4A–D); (5) bufalin increased the activities of caspase-3, -9, and -8 (Figure 5A–C); (6) bufalin increased pro-apoptotic proteins such as Bax, AIF, and Endo G (Figure 6A) and decreased anti-apoptotic proteins such as Bcl-2 and Bcl-x (Figure 6A); (7) bufalin induced endoplasmic reticulum stress associated with protein expression such as ATF-6α, caspase-4, GRP-78, and GADD153 (Figure 6C); (8) and bufalin increased Endo G (Figure 7A), cytochrome c (Figure 7B), and AIF (Figure 7C) protein release from mitochondria to cytoplasm.

Our results have shown that bufalin decreased the viable cell number dose- and time-dependently (Figure 1), which is in agreement with other reports from many other cell lines. In the cell cycle analysis, U-2 OS cells were analyzed by flow cytometry, and the results indicated that bufalin increased the G0/G1 phase accumulation time-dependently. This is in agreement with other reports, which showed that bufalin induced cell cycle arrest in many cancer cell lines such as gastric cancer cells, leukemia cells, and bladder carcinoma cells [23,33,34,35]. We have used Annexin V/PI double staining and TUNNEL assay for confirming bufalin induced cell apoptosis in U-2 OS cells. We found that bufalin significantly induced apoptotic cell death in U-2 OS cells, and these effects are time-dependent. It is well documented that Annexin V/PI double staining has been used as protocol for measuring apoptotic cell death (apoptosis) [36,37].

We have found that bufalin increased Ca^2+^ production and decreased the production of ROS and levels of ΔΨ_m_ in U-2 OS cells, and these effects are time-dependent (Figure 4). Numerous studies reported that cancer cells produce higher levels of ROS than do normal cells [38,39] and ROS play an important role in cancer progression by stimulating cell growth and genetic instability. ROS plays a key role in cancer cell death, under starvation or stress conditions, and ROS is increased for the induction of autophagy [40]. Furthermore, a chemotherapeutic agent induced necrotic or apoptotic cell death of cancer cells via the generation of ROS [41]. Therefore, we suggest that bufalin induced apoptotic cell death did not involve ROS production. Figure 4C clearly demonstrated that bufalin increased the Ca^2+^ production in U-2 OS cells. Ca^2+^ uptake into the mitochondrial matrix is critical for cellular function [42]. Various environmental signals may disrupt endoplasmic reticulum (ER) homeostasis to cause protein misfolding, and accumulation is called ER stress [43]. Herein, we also found that bufalin significantly increases the protein expression of ATF-6α, caspase-4, GRP-78, and GADD153 (Figure 6C), which are the hallmarkers of ER stress; thus, we suggest that bufalin induced cell apoptosis in U-2 OS cells through ER stress. Figure 4D also showed that bufalin suppressed the levels of ΔΨ_m_ in U-2 OS cells. Thus, our data indicate that bufalin exert a similar effect, as they increase mitochondrial oxidative stress, which contributes to apoptotic cell death in cancer cells [44]. Other studies have shown that anticancer drugs induced cell apoptosis via the dysfunction of mitochondria or decreased the levels of ΔΨ_m_ [37,45]. It was reported that both ROS and mitochondria are involved in the stimulation of apoptosis in the intrinsic signaling pathway [46,47]. Based on these observations, we may suggest that bufalin induced cell apoptosis of U-2 OS is through mitochondria-dependent pathway.

There are two main apoptosis pathways such as the death receptor (extrinsic) pathway and the mitochondrial (intrinsic) pathway [48,49]. The former is medicated via Fas and Fas-L (Figure 6B) involvement and caspase-8 activation, which can be triggered by cytotoxic stresses, and activates caspase-9 and -3 for induced cell apoptosis [48,49]. Thus, it is well known that cell apoptosis also can be divided between caspase-dependent and -independent pathways and activations of caspase-8 and caspase-9 that trigger the extrinsic and intrinsic cell apoptotic pathways [50,51]. The Caspase family, the specific apoptotic signal transduction molecular, is considered to be one of the most critical elements in the mechanisms of apoptosis [52]. In the present study, the results revealed that bufalin significantly increased caspase-8, -9, and -3 activities (Figure 5A–C). Thus, the activation of caspase-8, along with caspase-9 and -3, shows involvement of caspases in cell apoptosis in U-2 OS cells. We also used Western blotting to confirm that bufalin increased the expression of cleaved caspase-3, -8, and -9 proteins, and these effects are in a dose-dependent manner (Figure 6B). Mitochondrial control of apoptosis has been recognized to be the main mitochondrial membrane potential and membrane permeability [49]. Thus, after decreasing the level of ΔΨ_m_, it may lead to cytochrome c or/and AIF or/and Endo G release from mitochondria [49]. Herein, we also found that bufalin inhibited the expression of anti-apoptotic proteins such as Bcl-2 and Bcl-x and increased the pro-apoptotic proteins such as Bax in U-2 OS cells (Figure 6A). Furthermore, the results also showed that bufalin increased the expression of cytochrome c (Figure 6A) and the cleavage of PARP (Figure 6B) in U-2 OS cells. It is well documented that the ratio of Bax/Bcl-2 are involved in the levels of ΔΨ_m_, which is associated with the release of cytochrome c, AIF, and Endo G [49]. Herein, the alterations of the Bax/Bcl-2 ratio, which led to the dysfunction of mitochondria and then the release of cytochrome c (Figure 6A), followed the activations of caspase-9 and caspase-3, leading to apoptosis in U-2 OS cells. Furthermore, the results from western blotting also showed that bufalin increased the expression of AIF, cytochrome c, and Endo G (Figure 6A), which was confirmed by confocal laser system microscopy (Figure 7A–C).

In conclusion, we have investigated the cytotoxic effects of bufalin on U-2 OS human osteosarcoma cancer cells in vitro, and the results showed that bufalin induced cell morphological changes, decreased the total percentage of viable cells, and induced cell cycle arrest and cell apoptosis. Flow cytometry assays showed that bufalin increased Ca^2+^ productions and decreased the levels of ROS and mitochondria membrane potential, leading to cytochrome c, AIF, and Endo G release from mitochondria. Thus, we suggest that bufalin induced cell apoptosis via the extrinsic pathway, which involves Fas, Fas-L, and caspases (caspase-8), and also via intrinsic pathways, leading to AIF and Endo G release and inducing apoptosis; this is summarized in Figure 8.

## 4. Materials and Methods

### 4.1. Chemicals and Reagents

Bufalin of 99% purity, dimethyl sulfoxide (DMSO), 4′,6-Diamidino-2-Phenylindole, Dilactate, (DAPI), propidium iodide (PI), and Trypsin-EDTA were obtained from Sigma Chemical Co. (St. Louis, MO, USA). McCoy’s 5A medium, fetal bovine serum (FBS), l-glutamine, and penicillin-streptomycin were purchased from GIBCO^®^/Invitrogen Life Technologies (Carlsbad, CA, USA). Primary antibody anti-β-actin was purchased from Sigma-Aldrich (St. Louis, MO, USA); anti-Bcl-2, Bcl-x, Bax, caspase-3, caspase-8, caspase-9, and PARP were purchased from Cell Signaling Technology (Beverly, MA, USA); anti-cytochrome c, AIF, Endo G, Calpain 1, GRP-78, and GADD153 were purchased from Santa Cruz (Santa Cruz, CA, USA); anti-Fas and caspase-4 were purchased from BD Biosciences (San Diego, CA, USA); and anti-Fas-Ligand was purchased from Millipore (Bedford, MA, USA). Second antibody goat anti-mouse IgG-HRP, goat anti-rabbit IgG-HRP, and goat anti-goat IgG-HRP were purchased from Santa Cruz (Santa Cruz, CA, USA). Second antibody FITC (fluorescein isothiocyanate)-labeled goat anti–mouse IgG Ab, FITC-labeled goat anti-rabbit IgG Ab, and FITC-labeled goat anti-goat IgG Ab were purchased from Jackson ImmunoResearch (West Grove, PA, USA). Bufalin was dissolved in DMSO.

### 4.2. Cell Culture

The U-2 OS human osteosarcoma cell line was obtained from the Food Industry Research and Development Institute (Hsinchu, Taiwan). The cells were placed in 90% McCoy’s 5A medium with 10% fetal bovine serum (FBS), 100 units/mL penicillin, 100 μg/mL streptomycin, and 2 mM glutamine and were cultured in a humidified 5% CO_2_ incubator at 37 °C [53,54].

### 4.3. Cell’s Morphology Examination and Total Viability Assays

U-2 OS cells (1 × 10^5^ cells/well) were cultured in 12-well plates with McCoy’s 5A for 24 h and then were incubated with bufalin at final concentrations (0, 100, 200, 400, 600, and 800 nM) in each well or with 1% DMSO as a vehicle control for 24 and 48 h. For cell morphological change measurements, the cells were examined and photographed under contrast phase microscopy at ×200. For total viability measurements, cells were collected, counted, and stained with PI (5 μg/mL), followed by being immediately analyzed with flow cytometry (BD Biosciences, FACSCalibur, San Jose, CA, USA) for the viable cell number, as previously described [55].

### 4.4. Cell Cycle Distribution

Cell cycle distribution was examined and quantified by flow cytometry, as previously described [47]. Briefly, U-2 OS cells (1 × 10^5^ cells/well) were cultured in 12-well plates and were incubated with bufalin (200 nM) for 0, 12, 24, and 48 h, and then harvested and washed with PBS (phosphate-buffered saline) and fixed in 70% ethanol for 30 min in the dark at 37 °C with a solution containing 50 mg/mL PI and 50 μg/mL RNase A. Cells were analyzed for cell cycle distribution by FACSCalibur flow cytometer (Becton Dickinson). The percentage of G0/G1, S, and G2/M phase was assessed based on histograms generated by the CellQuest and Mod Fit computer programs (BD Biosciences Clontech, Palo Alto, CA, USA), as described previously [56].

### 4.5. Annexin V/PI Staining for Apoptotic Cell Death

An Annexin V-FITC apoptosis detection kit was used for measuring apoptotic cell death in a dual-staining protocol in which the apoptotic cells are stained with Annexin-V (green fluorescence) and the necrotic cells are stained with propidium iodide (PI) (red fluorescence), as described previously [49]. Briefly, U-2 OS cells (1 × 10^5^ cells/well) were cultured in 12-well culture plates and were incubated with 200 nM bufalin for 0, 12, 24, and 48 h. Cells were isolated and stained with Annexin-V/PI in the dark for 15 min, and the cells were re-suspended in Annexin-V binding buffer, followed by being immediately analyzed by flow cytometry (BD Biosciences, FACSCalibur, San Jose, CA, USA) [57]. Each experiment was done in triplicate.

### 4.6. TUNNEL Assay

Cell apoptosis also was examined by TUNNEL assay. U-2 OS cells (1.5 × 10^5^ cells/well) were cultured in 6-well culture plates and were incubated with 200 nM bufalin for various time periods. Cells were washed and were stained using the ApoBrdU DNA Fragmentation Assay Kit (BioVision, Mountain View, CA, USA) according to the manufacturer’s protocol, and apoptosis was observed using confocal laser scanning microscopy (TCS SP2, Leica, Wetzler, Germany) [58].

### 4.7. Measurements of Reactive Oxygen Species (ROS), Intracellular Ca^2+^ and Mitochondrial Membrane Potential

Flow cytometric assay was used to measure the levels of ROS, Ca^2+^, and ΔΨ_m_ in U-2 OS cells after being exposed to bufalin. Briefly, the U-2 OS cells (1 × 10^5^ cells/well) in the 12-well plates were incubated with bufalin (200 nM) for 0, 2, 6, 12, 24, and 48 h. For the measurement of ROS (H_2_O_2_), cells were isolated to be re-suspended with 500 μL of DCFH-DA (2′,7′-Dichlorofluorescin diacetate) (10 μM). For the measurement of the levels of ΔΨ_m_, cells were re-suspended with 500 μL of DiOC_6_ (4 μmol/L), and for the measurement of intracellular Ca^2+^ concentrations, cells were re-suspended with 500 μL of Fluo-3/AM (2.5 μg/mL), and then all samples were maintained in the dark for 30 min at 37 °C. After incubation, all samples were analyzed by flow cytometry, as described previously [59,60].

### 4.8. Measurements of Caspase-3, Caspase-8 and Caspase-9 Activities

Flow cytometry was used to measure the activities of caspase-3, -8m and -9 in U-2 OS cells after they were exposed to bufalin. Briefly, U-2 OS cells (1 × 10^5^ cells/well) were cultured onto 12-well plates and were incubated with 200 nM of bufalin for 0, 12, 24, and 48 h, and then cells were harvested and re-suspended in 25 μL of 10 μM substrate solution (PhiPhiLux-G1D1 for caspase-3, CaspaLux8-L_1_D_2_ for caspase-8 and CaspaLux9-M1D2 for caspase-9) and incubated at 37 °C for 60 min. After incubations, all samples were harvested, washed with PBS, and analyzed by flow cytometry for caspase-3, -8, and -9 activities, as described previously [59].

### 4.9. Western Blotting Analysis

U-2 OS cells (1.5 × 10^6^ cells) were cultured onto a 10 cm dish for 24 h and then were incubated with 200 nM bufalin for 0, 6, 12, 24, and 48 h. Cells were lysed and the total protein was measured by a Bio-Rad protein assay kit (Hercules, CA, USA) The 30 μg of protein extracts were separated by SDS-PAGE and transferred onto polyvinylidene [57], difluoride (PVDF) membranes (Millipore), and blocking, and then the membranes were hybridized with the primary antibodies anti-Bcl-2, -Bcl-x, -Bax, -Cytochrome c, -AIF, -Endo G, -caspase-3, -8, and -9, -Fas-L, -Fas, -PARP, -Calpain 1, -ATF-6α, -caspase-4, -GRP-78; GADD153; and β-actin overnight at 4 °C. Then the samples were washed and the membrane was incubated with peroxidase-conjugated anti-mouse, -rabbit, and -goat IgG (immunoglobulin G) (Santa Cruz Biotechnology) at room temperature for 1 h. Subsequently, the proteins were visualized, and chemiluminescence signals were enhanced using ECL (electrochemiluminescence) detection (Millipore) [61,62,63].

### 4.10. Confocal Laser Scanning Microscopy Assay

For examining the expression and translocation apoptotic associated protein that was performed by confocal system microscopy, U-2 OS cells (1.5 × 10^5^ cells/well) were placed on a 6-well plate, were incubated with bufalin (200 nM) for 48 h, and were fixed with 4% formaldehyde in PBS for 15 min. For 1 h, 0.1% Triton-X 100 in PBS was added to cells permeate the cells for blocking non-specific binding sites by using 2% BSA (bovine serum albumin). Primary antibodies anti-AIF, anti-Cyto c, and anti-Endo G were added to the cells overnight and were afterwards stained with a secondary antibody (FITC-conjugated goat anti-mouse, -rabbit, or -goat IgG) and PI (red fluorescence) for nuclei examination, as described previously [59]. Slides were mounted, examined, and photo-micrographed under a Leica TCS SP2 Confocal Spectral Microscope (Leica Microsystems, Heidelberg, Mannheim, Germany).

### 4.11. Statistical Analysis

The data were presented as the mean ± standard deviations (SD) from three independent experiments. Differences in statistical significance between the values of the control and bufalin treated groups were analyzed by Student’s *t* test. Statistical significance was considered when the P value was less than 0.05 for time- and dose-dependent assay, The values were analyzed by one-way analysis of variance (ANOVA), followed by Duncan’s multiple range test (DMRT).
